# Supplementary material for: Understanding Quality of Pinot Noir Wine: Can Modelling and Machine Learning Pave the Way?
Source: Foods. 2022 Oct 3;11(19):3072. doi: 10.3390/foods11193072 (PMC9562230; doi:10.3390/foods11193072)
Supplement: Supplementary file 1 [file foods-11-03072-s001.zip › foods-1905684-supplementary.pdf]

Supplementary Information

S1. Buckingham's Pi-theorem:

Let $x_1, x_2, \dots \dots, x_n$ be n dimensional variables which are physically relevant and related to a given problem. An unknown dimensionally homogenous set of equations connects these n dimensional variables that is physically significant in a given scenario.

These variables can be expressed using a functional relationship defined as

$$F(x_1, x_2, \dots \dots, x_n) = 0$$

or

$$x_1 = f(x_2, \dots \dots, x_n)$$

There will be k primary variables required to explain these n variables, and the other variables can be written as $(n - k)$ dimensionless and independent quantities known as 'Pi-groups,' As a result, the functional connection can be simplified to a far more compact form. It's important to note that this set of non-dimensional characteristics isn't unique. They are, nevertheless, self-contained and make up a full set. Even if the exact set of governing equations is unknown, the Buckingham-Pi theorem describes a method for computing dimensionless parameters from given variables.

Let us consider that there are $n = 3$ dimensional quantities to consider say mass, length and time. Suppose the model involves $m = 6$ variables, denoted $x_1, x_2, \dots \dots, x_6$. In general, we can derive $m - n$ e.g., 3 dimensionless groups, often denoted as π -groups using the following steps:

- Determine the dimensions for all variables $x_1, x_2, \dots \dots, x_6$
- Select n of the variables - say $x_1, x_2,$ and x_3. These n variables are called the repeating variables and will appear in all the π terms.
- Now choose one other variable - say x_4. Some combination of x_1, x_2, x_3 and x_4 is dimensionless and forms the first π term or dimensionless group.
- Now use x_1, x_2, x_3 and x_5 and repeat the process until there are no more variables left.
- Once we figure out all the dimensionless groups, we can describe the relationship between the variables as a relationship between the various groups.

  Note: We have to deal with certain constraints on the choice of variables:

  - ⊙ Recurring variables cannot be dimensionless.
  - ⊙ The total dimension of two recurring variables cannot be the same.
  - ⊙ Buckingham's theorem works as a formula for calculating sets of dimensionless numbers, but it doesn't state anything about their physical significance, though.

⊙ Non-dimensional groups can adopt any functional structure once they have been determined.

The Buckingham-Pi theorem is not limited to a specific field and gives a mathematical framework for creating non-dimensional groups for a physical quantity. To interpret and appropriately utilise non-dimensional groups in a subsequent examination of any model, we need to understand the physics of the underlying problem.

Table S1. Descriptive statistics of data1

Chemical Compound	Mean	Std	Min	First Quartile	Median	Third Quartile	max
Ethyl acetate	118752.9	21349.67	89839.04	101647.6	113534.7	136361.2	167570.1
Ethyl 2-methylpropanoate	243.5829	84.99485	145.1913	182.9255	232.0147	265.3345	416.2976
Ethyl butanoate	280.5432	80.89879	186.1078	229.3697	273.9664	306.2146	544.9659
Ethyl 3-methylbutanoate	35.95041	12.94038	15.2404	28.58317	33.3496	39.86595	64.1671
2-Methylpropan-1-ol	61148.66	8743.97	36696.52	56818.85	60666.13	67206.51	74067.52
3-Methylbutyl acetate	160.5069	42.79374	112.1783	132.0359	146.6869	185.9975	278.792
Ethyl pentanoate	1.473361	0.521735	0.9333	1.113375	1.25035	1.8235	2.7772
3-Methylbutan-1-ol	174224.5	29671.59	141578.2	152023.8	165589.5	181570.9	238397.1
Ethyl hexanoate	437.1696	79.58736	318.5636	404.3063	422.5062	480.298	666.3077
Hexyl Acetate	5.745911	6.926808	1.4614	2.471325	3.0604	6.848425	31.9158
Ethyl 2-hydroxypropanoate	171857.2	31976.94	125956.6	148126.3	162451.5	191605.1	233843.2
Hexan-1-ol	2104.15	431.7344	1421.54	1809.077	2024.099	2255.133	3051.925
(E)-Hex-3-en-1-ol	77.02175	13.3867	48.9605	69.40158	75.5357	81.10418	102.2955
Ethyl heptanoate	2.029622	0.378561	1.4039	1.817275	2.0112	2.196775	3.0845
(Z)-Hex-3-en-1-ol	45.7253	19.23528	24.0479	38.87183	41.4003	50.13237	112.4569
Heptan-1-ol	43.32062	8.036723	26.288	39.2958	42.8614	47.29363	56.8897
Ethyl octanoate	575.3734	95.35276	389.6934	516.971	592.0891	643.6546	721.3681
Benzaldehyde	14.14813	13.38348	0.1657	4.100625	11.48415	17.82288	53.6889
Ethyl decanoate	274.8226	101.5478	65.7652	207.8458	262.4152	341.4423	461.8009
2-Phenylethan-1-ol	36241.13	18696.78	23003.95	25283.02	30544.65	37095.61	101412.6
2-Methylpropyl acetate	73.18496	21.40754	42.7031	58.8575	67.1782	80.7205	119.9842
Ethyl 2-methylbutanoate	31.79282	9.718278	19.4123	26.34065	30.0029	34.45715	51.8135
2-Methyl butyl acetate	253.6515	71.86906	163.1023	207.3275	229.2287	287.9782	462.3387
(E)-Hex-2-en-1-ol	15.25405	7.5614	5.3945	9.313375	14.45435	18.7158	30.4454
3,7-Dimethylocta-1,6-dien-3-ol	4.112922	1.583573	2.2897	3.380725	3.804	4.190625	9.4554
Octan-1-ol	67.23637	16.1202	31.7351	57.51248	67.7675	77.57065	99.1281
3,7-Dimethyloct-6-en-1-ol	4.235667	1.570415	1.4807	3.248675	4.1157	5.4845	7.3192
(2Z)-3,7-Dimethylocta-2,6-dien-1-ol	2.642422	0.951269	1.7874	1.950875	2.4888	2.86665	5.6756
2-Phenethyl acetate	19.83248	11.12106	9.8963	13.5586	15.52795	20.4052	55.3471
(E)-1-(2,6,6-Trimethylcyclohexa-1, 3-dien-1-yl) but-2-en-1-one	1.834239	0.551174	0.9183	1.470525	1.83145	2.01625	3.1174
2-Methoxyphenol	10.76286	3.364831	6.4268	8.416025	10.2497	12.23103	19.4331
(2E)-3,7-Dimethylocta-2,6-dien-1-ol	4.229306	3.6886	1.258	2.229225	2.63435	4.604725	14.4553
(E)-4-(2,6,6-Trimethylcyclohex-2-en-1-yl) but-3-en-2-one	0.0605	0.008415	0.0481	0.055525	0.06035	0.063775	0.08
Ethyl 3-phenylpropanoate	1.310433	0.644821	0.5328	0.8545	1.0302	1.66715	3.1082
(E)-4-(2,6,6-Trimethylcyclohexen-1-yl)	1.360783	0.103078	1.1948	1.276375	1.37905	1.4196	1.5425

but-3-en-2-one							
Phenol	8.232989	2.009847	5.9453	6.723425	7.6179	9.285425	13.011
4-Ethyl-2-methoxyphenol	3.113858	4.03652	0.5848	0.932	1.22235	3.5254	15.0148
Ethyl (E)-3-phenylprop-2-enoate	3.76615	2.719918	1.2295	2.0104	2.3987	5.04705	11.1255
2-Methoxy-4-prop-2-enylphenol	24.18552	3.91557	18.9405	21.68312	22.86525	25.60648	32.9726
Methyl-2-aminobenzoate	3.839	1.404199	1.3255	2.97285	3.5465	5.108175	6.1837
Acetic acid	620964.6	102939.8	451071.7	555058.1	594726.7	698778.2	864620.6
2-Methylpropanoic acid	1898.17	669.7691	1227.35	1416.9	1854.848	2092.094	4175.823
Butanoic acid	1112.333	285.595	817.807	955.3066	1032.99	1170.22	2120.864

(Std≈standard deviation, min≈minimum, max ≈maximum)

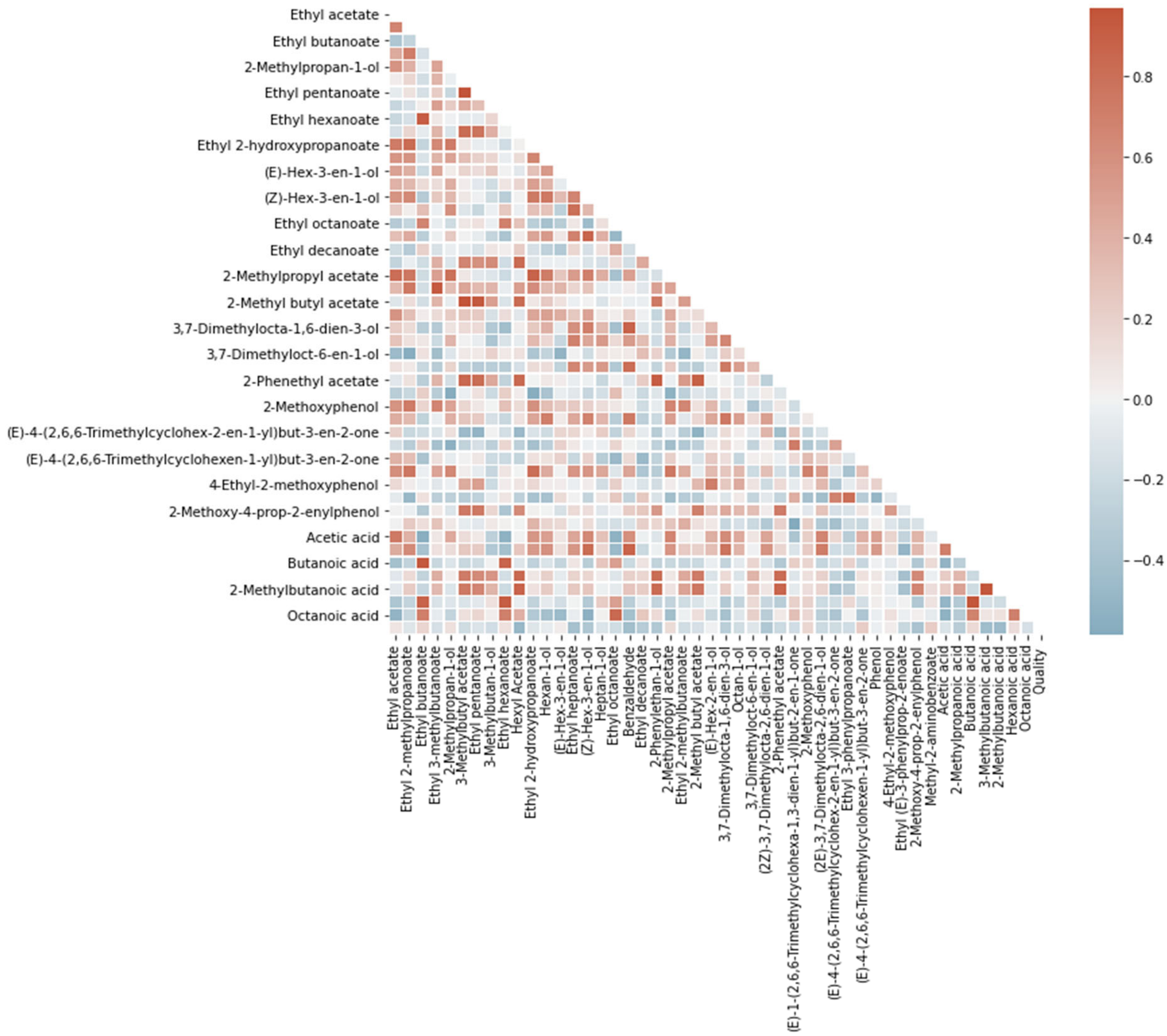

Figure S1. Correlation among aroma compounds using Pearson correlation method

Table S2. Descriptive statistics of data2

Features	count	mean	std	min	25%	50%	75%	max
Ethanol	1000	13.76397	0.548334	12.26	13.56	13.95	14.06	14.78
pH	1000	3.64243	0.072847	3.43	3.6	3.66	3.7	3.77
Titratable acidity	1000	5.02108	0.216041	4.61	4.8375	5.09	5.18	5.55
Total Sulphur	1000	0.05232	0.014312	0.03	0.04	0.05	0.06	0.1
Reducing Sugars	1000	0.4201	0.115927	0.23	0.32	0.42	0.5	1.17
Total Phenolics	1000	1.70518	0.412594	1.06	1.29	1.73	1.9925	2.58

(TA stands for titrable acidity)

Table S3. Descriptive statistics of data3

Features	count	mean	std	min	25%	50%	75%	max
Titratable acidity	1000	5.28859	0.166021	4.89	5.2	5.29	5.3725	5.85
Total Sulphur	1000	0.04516	0.007657	0.03	0.04	0.04	0.05	0.06
Total Phenolics	1000	1.75465	0.473115	1.37	1.51	1.60	1.76	3.91
Reducing Sugars	1000	0.51487	0.407983	0-00	0.34	0.39	0.50	2.57
pH	1000	3.69401	0.054661	3.56	3.65	3.70	3.74	3.79
Ethanol	1000	13.69208	0.259770	13.25	13.49	13.72	13.87	14.27

Table S4: Physiochemical Dataset for case study 1 and case study 2. Standard parameters estimated for the 18 wines are coded as: RS = reducing sugars; TA = titratable acidity; Total phenolics = a measurement of the concentration of all phenolic material present in the wine and expressed as mg gallic acid equivalents/L

**Wine ID Set A**	**TA (g/L)**	**Total Sulphur (g/L)**	**Total phenolics (g GAE/L)**	**Reducing sugar(g/L)**	**pH**	**Ethanol (v/v%)**	**Wine ID Set B**	**TA (g/L)**	**Total Sulphur (g/L)**	**Total phenolics (g GAE/L)**	**Reducing sugar (g/L)**	**pH**	**Ethanol (v/v%)**
WAR16	5.17	0.0304	1.9172	0.53	3.6	14.78	OQRB	5.59	0.0649	1.435	0.29	3.77	14.27
MCH16	5.47	0.0448	2.4327	0.69	3.5	14.29	OGG	5.33	0.0439	1.416	0.37	3.7	13.53
WCR16	4.87	0.0336	2.4501	0.33	3.66	14.43	OFCP	4.89	0.0420	1.611	0.34	3.79	13.84
WE16	4.61	0.0496	2.2304	0.33	3.72	13.74	OWTB	5.47	0.0290	1.693	0.27	3.61	13.45
OFRB316	4.98	0.0576	1.6153	0.3	3.61	13.92	OM	5.34	0.0519	1.367	0.34	3.65	13.91
OFRCP16	5.36	0.0352	1.6565	0.48	3.53	14.32	OMCO	5.45	0.0290	1.795	2.57	3.72	13.68
MG16	5.55	0.0376	2.1550	0.3	3.52	14.26	MCHPC	5.3	0.0380	3.911	0.49	3.67	13.25
MG13	4.62	0.0496	1.3122	0.41	3.72	13.47	MGM	5.38	0.0439	1.559	1.13	3.72	13.76
CG16	4.63	0.0528	1.2880	0.32	3.73	13.99	MVA	5.27	0.0509	1.54	0.52	3.75	14.03
OMD16	5.04	0.0592	1.0576	0.47	3.43	12.75	MSCOR	5.25	0.0360	2.465	0.57	3.65	13.46
NN16	5.09	0.048	2.1465	0.23	3.67	13.97	MGC	5.13	0.0559	1.768	0.4	3.62	13.26
WPP16	5.19	0.04	1.7959	0.27	3.7	13.97	MA	5.85	0.0350	2.033	0	3.56	13.61
WPP13	4.7	0.08	1.6559	0.38	3.77	12.26	WTT	5.59	0.0330	1.42	0.33	3.67	13.5
CPB16	5.15	0.0512	2.5812	0.52	3.6	14.16	WCR	5.4	0.0619	1.419	0.5	3.71	12.88
MPR16	5.49	0.0528	2.0270	0.48	3.66	14.54	WCR	5.87	0.0509	3.465	0.6	3.79	14.51
OQR16	5.43	0.096	1.7850	0.54	3.55	13.36	WPL	4.73	0.0430	1.587	1.36	3.8	13.7
OQR13	4.95	0.0624	1.1163	0.69	3.65	13.83	WTKJM	5.72	0.0459	1.605	0.25	3.62	13.59
NS16	5.33	0.0512	1.3921	1.17	3.77	13.2	WAR	5.15	0.0479	1.808	0.55	3.8	13.62

S2. Deep Neural Network (DNN) in the present study:

A deep neural network (DNN) is a form of neural network that has multiple neural layers. The feed forwards DNN, also known as the multiple layer perceptron, was used in this research. Feed forwards DNN, as the name implies, is made up of numerous hidden layers that only move in the feed forwards direction (no loop back). Weights, biases, and non-linear activation are the most significant scenarios in the feed forwards DNN. The parameters that alter input data within the network's hidden layers are known as weight in any neural network. On the other hand, bias cam considered as the analogous to the role of a constant in a linear function, whereby the line is effectively transposed by the constant value. First layer is always the input layer, and we need to find one or more patterns from the entities of the input, so that those patterns can be used to achieve the output. To achieve that, we need number of hidden layers together with activation function. At the most basic level, an activation function decides whether a neuron should be fired or not. It accepts the weighted sum of the inputs and bias as input to any activation function. Step function, Sigmoid, RELU, Tanh, and Softmax are

examples of activation function. In order to achieve output, we need to adjust weights, biases such that, hidden nodes are activated by the activation function. Initially, weights and bias are randomly initialized. Then, we train the network with tens of thousands of inputs. In this study, DNN is used for three datasets (Data1, Data2, Data3) using 5 layers. Data1 contains input dimension of 14, on the other hand, Data2 and Data3 comprise of 6 input dimensions. In case of Data1, RELU function was used as the activation function followed by ELU in next three layers. Furthermore, 64 units were used in the first and second layer, however, units in dense layer 3 and 4 contains 64 and 32 units, respectively. The density layer of any neural network's dense layer determines the size of the dense layer's output. DNN with Data, on the other hand, has 64 units in the first three dense layers and 8 units in the fourth dense layer, with the RELU activation function utilised in all dense layers. Furthermore, the first two dense layers of DNN with Data 3 include 128 units, followed by 64 units in the third and fourth dense levels. In addition to that, similar activation function as in case of DNN with Data 1 were used. Finally, last layer of all three models consists of only one layer as output layer. We also used optimizers in this study such as RMSprop, and Adam. Optimizers in any DNN is necessary because it modifies the attributes of the network such as weights and learning rate. The RMSprop optimizer restricts the oscillations in the vertical direction. Therefore, we can increase our learning rate and our algorithm could take larger steps in the horizontal direction converging faster. On the other hand, ADAM combines the property of AdaGrad and RMSProp algorithms to provide an optimization algorithm that can handle sparse gradients on noisy problems. Additionally, DNN models were implemented at 2000 Epochs which represents one iteration over the entire dataset. However, early stopping was used to find the appropriate results at low epochs but not more than 2000 epochs. In other words, early stopping is the regularization technique that stops training if, for example, the validation loss reaches a certain threshold. After building and training of the DNN, there is always requirement of evaluators to judge the model performance, these evaluators also known as the loss metric and are Regression loss function, mean squared error, mean absolute error (MAE), and binary cross entropy loss. Present study is the regression-based study, hence, we used MAE as the loss metric to evaluate the performance of the model.

S3.

Pseudocode of the optimisation programme to calculate possible combination in order to get quality indices

Input:
N_d = the number of essential modulators;
N_f = the number of formulae;
N_b = the of bottles (18 bottles);
D[i][j] = null; //Variable for essential modulators
C[i][j][k] = null; //Variable for possible combination
Output: set of optimal values
Initialisation;

for *i* = 1 to N_d ***do***
    for *j* = 1 to N_f **do**
    D[i][j] = Calculation following formular of each modulator;
    end for i
    set *j* = 1;
end for j

while (! possible combination ended)
for *i* = 1 to N_d ***do***
    for *j* = 1 to N_f **do**
        for *k* = 1 to N_b **do**
        C[i][j][k] = calculating possible combination;
        end for k
        set *k* = 1;
    end for i
    set *j* = 1;
end for j
Record combination into the database;
end while

// selection of the optimal combination
Get data from database;
while (! possible combination ended)
    calculating distance between each combination and the experts' observations by Euclidian distance;
    update Euclidian distance value to the combination;
End while
ascending order of Euclidian distance value;
get the optimal values;
